# From ‘making lists’ to conducting ‘well-rounded’ studies: Epistemic re-orientations in soil microbial ecology

**DOI:** 10.1177/03063127231179700

**Published:** 2023-06-30

**Authors:** Ruth Falkenberg, Lisa Sigl, Maximilian Fochler

**Affiliations:** University of Vienna, Vienna, Austria

**Keywords:** epistemic re-orientation, do-ability, research practices, scientific innovation, research governance

## Abstract

Soil microbial ecology is a relatively young research field that became established around the middle of the 20th century and has grown considerably since then. We analyze two epistemic re-orientations in the field, asking how possibilities for creating do-able problems within current conditions of research governance and researchers’ collective sense-making about new, more desirable modes of research were intertwined in these developments. We show that a first re-orientation towards molecular omics studies was comparably straightforward to bring about, because it allowed researchers to gain resources for their work and to build careers—in other words, to create do-able problems. Yet, over time this mode of research developed into a scientific bandwagon from which researchers found it difficult to depart, even as they considered this kind of work as producing mostly descriptive studies rather than exploring interesting and important ecological questions. Researchers currently wish to re-orient their field again, towards a new mode of conducting ‘well-rounded’ interdisciplinary and ecologically-relevant studies. This re-orientation is, however, not easy to put into practice. In contrast to omics studies, this new mode of research does not easily enable the creation of do-able problems for two reasons. First, it is not as readily ‘packaged’ and hence more difficult to align with institutional and funding frameworks as well as with demands for productivity and career building. Second, while the first re-orientation was part of a broader exciting bandwagon across the life sciences and promised apparent discoveries, the current re-orientation goes along with a different sense of novelty, exploring complex environmental relations and building an understanding at the intersection of disciplines, instead of pushing a clearly circumscribed frontier. Ultimately, our analysis raises questions about whether current conditions of research governance structurally privilege particular kinds of scientific re-orientation over others.

From time to time, researchers may desire to re-orient the epistemological basis of their field, whether for the sake of scientific knowledge itself or to better position the field to address the problems of the time. This paper is concerned with these processes of change and what facilitates or impedes them. We explore two subsequent re-orientations in a research field and examine why the first was easier to bring about and why the second has been causing frictions. In describing and comparing these re-orientations, we focus on the role of possibilities and difficulties with creating do-able problems, thus analyzing the influence of current conditions of research governance on the epistemic direction a field takes (Gläser & Laudel, 2019; Whitley et al., 2018). However, we also consider what researchers, for reasons of both scientific and social-environmental relevance, collectively see to be desirable directions for their field. Hence, we underline that researchers are not only strategic actors who aim to construct do-able problems but they also bring other valuations to their work.

Our case, soil microbial ecology, is a relatively young field that became established in the second half of the twentieth century and has grown considerably since then (Caumette et al., 2015; Kowalchuk et al., 2007). This field aims to understand the mutual interactions of soil microbial communities with their biotic and abiotic environments (Hallin & Bodelier, 2020; Veldkamp, 1985). Soil microbial ecology is an especially interesting case because of different dynamics that influence researchers’ possibilities to make research problems do-able. The field is frequently described as highly relevant to current social-environmental problems, but also as strongly driven by innovation in research technologies and, related to its growth, subject to increasing competition. Furthermore, the complexity of soils generally makes it difficult to grasp the manifold interactions between microbial communities and their environment (Fierer, 2017; Lehmann et al., 2020). How to handle the complexity of soils both in research practices and in approaching social-environmental problems is much discussed in soil microbial ecology as well as in soil-related research more broadly (Puig de la Bellacasa, 2015; Sigl et al., 2023).

This paper is based on an analysis of soil microbial ecology literature, an ongoing in-depth engagement with one soil microbial ecology research group, interviews, and one group discussion with prominent scientists from the field. We show that a first re-orientation (roughly starting in the 1990s) towards molecular methods and omics techniques, although technologically challenging, was experienced by involved researchers as having been relatively straightforward to bring about because it soon allowed researchers to create do-able problems. Yet a second re-orientation that soil microbial ecologists currently desire is described as more difficult: one towards encompassing ‘well-rounded’ studies that combine various disciplinary and technological approaches. Many researchers argue that this more integrative mode of research is better suited for studying the ecological functions and complex interactions of microbial communities with particular soil habitats, and for increasing the field’s capacity to address social-environmental problems. However, in contrast to omics studies, this new mode of research does not easily lead into do-able problems: First, it is not as readily ‘packageable’ (Fujimura, 1987), which makes it more difficult to align with institutional and funding frameworks and a riskier bet when it comes to guaranteeing productivity and building careers. Second, while the first re-orientation was part of a broader exciting ‘bandwagon’ across the life sciences and clearly promised many discoveries (Fujimura, 1988), the second re-orientation comes with a different sense of novelty: exploring complex ecological interactions and building an understanding at the intersection of disciplines instead of pushing a clearly circumscribed frontier.

We thus show that while collective sense-making about new, more desirable modes of research can play an important role in processes of change in science, difficulties with turning a new mode of research into do-able problems can hinder or slow down epistemic re-orientation even if researchers collectively favor it. Our analysis indicates that, to some extent, current conditions of research governance privilege epistemic re-orientations that are more easily packageable and broadly seen as innovative, whereas re-orientations towards more integrative ways of knowledge production that lack such attributes may be impeded. We conclude that enabling research fields to re-orient themselves towards such more integrative modes of research—which are often seen as crucial for addressing pressing social-environmental crises not only on soil microbial ecology, but also in, for example, the broader field of ecology (Carpenter et al., 2009; Hampton & Parker, 2011) or soil science (Sigl et al., 2023)—will require building respective enabling conditions within the research ecosystem.

## Capturing change towards new modes of research

In the literature broadly concerned with change in science, the emergence of new research fields has been much discussed by both older (Ben-David & Collins, 1966; Edge & Mulkay, 1976; Law, 1973; Mullins, 1972) and more recent studies (Merz & Sormani, 2016; Molyneux-Hodgson & Meyer, 2009; Vermeulen, 2018). Changes in the epistemic orientation of existing research fields or disciplines, however, are not always easy to grasp. Such processes have been discussed under various labels, ranging from ‘paradigm shifts’ (Kuhn, 1970) to ‘scientific innovation’ (Gläser & Laudel, 2019; M. J. Mulkay, 1970). Since Kuhn (1963), different authors have pointed to the ‘essential tension’ between the quest for new knowledge and intellectual breakthroughs, and the tenacity of established views (see e.g. M. Mulkay, 1972; Philipps & Weißenborn, 2019; Siler & Strang, 2017). This underlines the importance of considering why and under what conditions scientific re-orientations—in the sense of a large number of researchers changing their epistemic orientation and respective research practices—can take hold in a field. Studies have analyzed different aspects that may play a role in such change, including steering by external actors such as industry or military (Barth, 2003; Doel, 2003), social-environmental challenges such as the climate crisis (Hirsch & Long, 2021), or innovation in research technologies and the creation of ‘standardized packages’ (Fujimura, 1988). Of particular interest to our endeavor are approaches that have considered the links between changes in the epistemic orientation of disciplines and conditions of do-ability in the research system, highlighting that possibilities for creating do-able problems can play a crucial role in facilitating scientific change (Fujimura, 1987, 1988; see also Gläser & Laudel, 2019; Whitley et al., 2018).

In STS and adjacent fields such as the history of science, re-orientations and processes of change in science have been discussed under various conceptual labels. On the level of science as a whole, Gibbons et al. (1994) have diagnosed their much-discussed *Mode 2* of knowledge production as a widespread change to more integrated and societally embedded ways of research. Another example is provided by Pestre (2003, p. 246), who rather sees science as having changed along different ‘heterogeneous *regimes* of knowledge production,’ highlighting the role that an embedding in particular political economies plays in such change processes. In this paper, we are less interested in how science changes as a whole than we are in re-orientations of particular fields. While Kuhn’s notion of *paradigm shifts/scientific revolutions* laid important ground in this regard, one of the critiques voiced towards his account is that it focuses too strongly on the role of theoretical developments in scientific change (Ankeny & Leonelli, 2016). More recent work has suggested different concepts for capturing not only epistemic and theoretical developments but also their intimate entanglement with organizational, material, technological, and institutional aspects of scientific change. Focusing on the biomedical realm, for example, Keating and Cambrosio (2003, p. 47) describe the emergence of *biomedical platforms* as a new mode of conducting research and of aligning biology and medicine, coming about through ‘the creation of a new epistemological, institutional, and material configuration.’ In a later book, the same authors (Keating & Cambrosio, 2014) analyze the rise of clinical cancer trials as a new *style of practice*, a notion that likewise highlights the intertwinement of institutions, scientific practices, ideas, and technologies in processes of scientific change. Another more recent suggestion for capturing change in science comes from Ankeny and Leonelli (2016, p. 18), who formulate the notion of *research repertoires* as ‘well-aligned assemblages of the skills, behaviours, and material, social, and epistemic components that a group may use to practice certain kinds of science.’ Particularly interesting about the latter approach is how Ankeny and Leonelli (2016, p. 20) articulate the notion of research repertoires with respect to ‘decisions and strategies concerning funding’ and the ‘management and dissemination of resources and outputs,’ both of which are crucial for change in science.

Research repertoires (Ankeny & Leonelli, 2016) and styles of practice (Keating & Cambrosio, 2014) provide conceptualisations that are sensitive to the complex intertwinements of different aspects of change in science and to how certain kinds of research can stabilize. However, their emphasis is less on unpacking these intertwinements by studying the work and decisions of individual researchers or groups. Our argument in this paper is that to understand the dynamics of both unsuccessful and successful re-orientations in science, it is crucial to attend to them through the lense of research practices, and more precisely the relation and potential tensions between what researchers aim to achieve (i.e., the direction they want to give their work and their field) and what they assess to be able to achieve given the technological, epistemic and institutional possibilities they are situated in.

In this regard, it is valuable to go back to the work of Fujimura (1987) on do-ability. Fujimura (1988, p. 261) emphasizes that change in science is ‘inseparable from both the local and broad scale organization of work and technical infrastructures’ and that such change is only likely to be successful when it allows scientists to construct *do-able problems*. For Fujimura (1987), the do-ability of (new) research problems or agendas depends not only on ‘production work,’ such as carrying out a relatively clearly circumscribed experiment, but also on ‘the alignment of several levels of work organization’; in particular, the levels of the experiment, the laboratory, and the ‘social world’ (p. 258). For example, scientists must align their choice of research problems with the conditions of available funding or convince colleagues in the field of the value of their work and findings. In turn—and this is our approach in this paper—empirical attention to how researchers construct their problems, value the desirability of addressing them, and assess their do-ability in a given context is necessary to understand how epistemic shifts are realized, inhibited or fail.

## Re-considering do-able problems in scientific change

We assume with Fujimura that constructing do-able problems plays a crucial role in scientific change, but we find it useful to adapt and re-articulate her conceptual approach to better grasp processes of change in contemporary research systems. We therefore explicitly distinguish two temporal dimensions that matter in researchers’ assessments of whether certain research problems are do-able. First, we see do-ability as shaped by whether the material, human, and temporal resources for conducting particular research projects are available or attainable in the present. In the current research landscape, where many research groups lack stable base funding, these resources increasingly need to be covered by competitive project funding (Franssen & De Rijcke, 2019). Hence, do-able problems usually need to fit with given funding opportunities. This fit may include their size and the degree of collaboration they involve, but also their specific temporalities that require the construction of ‘knowledge-time packages’ (Felt, 2017, p. 136), which are do-able within certain timeframes (Ylijoki, 2016).

Second, we see projections of possible futures—that is, assessments of the ‘riskiness’ or ‘safety’ of a research endeavor, its potential for productivity in terms of publishable output within given timeframes, and considerations about what is required to remain competitive and build successful careers in science—as essential to constructing do-able problems in contemporary academia (see Fochler & Sigl, 2018). While it might be argued that these aspects do not directly impinge on do-ability in the present, previous research has shown that such considerations can acquire strong formative power in decision-making processes, especially for junior researchers (Fochler et al., 2016; Müller, 2014). Put differently, even if a project may be do-able in the present, researchers may consider it non-do-able because of its likely impact on their future careers.

In addition to these temporal dimensions, we emphasize the importance of certain cross-cutting aspects which facilitate the construction of do-able problems—namely, the packageability of a certain mode of research and its potential to signal innovativeness and novelty. The first of these aspects has been discussed by Fujimura. She notes that when a particular approach to a research problem reaches a certain degree of standardization—coming as a readily available package of theory and method that provides researchers with a ‘clearly defined set of conventions for action that helps reduce reliance on discretion and trial-and-error procedures’ (Fujimura, 1988, p. 261)—this greatly facilitates the construction of do-able problems. Notably, it does so along both of the temporal dimensions sketched above: A sufficiently packaged approach is more likely to be supported by funding agencies, which enables researchers to attain resources in the present. It can also help scientists remain competitive and build successful careers by facilitating productivity and the attainment of jobs. If such an approach broadly enables scientists to construct do-able problems, it may turn into a scientific bandwagon: a mode of research to which ‘large numbers of people, laboratories, and organizations commit their resources’ (Fujimura, 1988, p. 261), which can bring about scientific change at a broader scale.

The second aspect that we see as crucial in facilitating do-ability is the degree to which a mode of research can be articulated as innovative and novel—its potential to function as an ‘accelerant of excitement’ in the ‘emotional cultures of science’ (Pickersgill, 2021, p. 613). In the current research landscape, scientists increasingly need to demonstrate the novel and innovative character of their work in order to acquire funding. Promising exciting discoveries and being able to work with the latest technologies in their field also play a crucial role in getting published and building a successful career in science (Falkenberg, 2021; Falkenberg & Fochler, 2022). While the importance of ‘sexy’ approaches that aid the construction of do-able problems has also been mentioned by Fujimura (1988), we argue that this aspect has become considerably more important to consider in studying contemporary academia.

Drawing on this conceptualization, we analyze the role of possibilities or difficulties with constructing do-able problems in two subsequent re-orientations in soil microbial ecology. At the same time, we emphasize that constructing do-able problems, and thereby remaining competitive is only one way in which scientists evaluate the choice of research problems and approaches. While this way of valuing research is becoming increasingly dominant in contemporary academia, it is nevertheless always negotiated alongside other evaluative principles (see, e.g., Fochler et al., 2016). Researchers may also desire to shift the epistemic orientation of their work even at the risk of competitive disadvantage, for example, because they believe this to be important to better address social-environmental problems or because they consider it necessary to advance their field scientifically. In our paper, we emphasize the importance of taking into account not only considerations about do-ability, but also such other valuations that researchers bring to their work (cf., Schikowitz, 2020). We illustrate that valuations of scientific and social-environmental relevance play an important role in processes of collective sense-making about the desirability of epistemic re-orientation in soil microbial ecology. However, we also show that although researchers may collectively favor a new mode of research for reasons of scientific and social-environmental relevance, epistemic re-orientation can be hindered by structural difficulties to turn a new mode of research into do-able problems.

## Omics and data-driven research as a scientific bandwagon

To better understand the first re-orientation in soil microbial ecology and parts of the difficulties that researchers currently see with again re-orienting their field, it is important to put these developments in the context of the prominence of (big) data-driven science and the rise of various omics approaches since the 2000s. As is now widely acknowledged, the ‘genomics revolution’ with the Human Genome Project as its centerpiece had transformative character across the life sciences (Hilgartner, 2017). While much research in STS and adjacent fields has focused on the political, social, and ethical implications of genomics research (Gibbon et al., 2018; Rajan, 2006; Rose, 2007), another body of work has investigated related transformations of concepts, practices, and research agendas within the life sciences (Hilgartner, 2017; Richardson & Stevens, 2015; Stevens, 2011). Such studies have illustrated that, following the Human Genome Project, much research in the life sciences has been centered around further developments in high-throughput technologies and the gathering of omics data (genomics, transcriptomics, proteomics, etc.)—a new mode of research frequently denoted as ‘post-genomics’ (Morange, 2006; Rheinberger, 2008; Rheinberger & Müller-Wille, 2018; Richardson & Stevens, 2015). Post-genomic research is sometimes described as being primarily focused on data work rather than on other forms of scientific creativity or innovation (Richardson & Stevens, 2015; Stevens, 2011). As put by Stevens (2011), the new ‘hyper-productive, “bioinformatic” biology—based on computers, automation and high-throughput—[is] oriented towards large volumes of data, towards speed’ (p. 218); it entails both changing notions of valuable work in biology and changes in the kinds of knowledge that are produced. While other authors have cautioned against seeing current data-driven science as an entirely new way of doing research (Bonde Thylstrup et al., 2019; Leonelli, 2016, 2019; Strasser, 2019), what certainly seems to deserve attention is the ‘prominence and status acquired by data as commodity and recognized output’ (Leonelli, 2014, p. 1) in the contemporary life sciences.

Adding to previous historical (Strasser, 2019) or philosophical (Dupré & O’Malley, 2007; Leonelli, 2016) angles on post-genomics and data-driven science, we focus on the relation of omics and data-intensive research to the social and institutional workings of current science. From this perspective, we understand big data and omics technologies as a scientific bandwagon across the life sciences and, more specifically, within soil microbial ecology. We suggest that in the context of wider excitement about (post)genomics, increased appreciation of data-intensive research, and institutional support for such modes of research, high-throughput sequencing and subsequent omics techniques provided soil microbial ecologists with an approach that was both packageable and innovative, which helped them construct do-able problems. This facilitated a first re-orientation of soil microbial ecology in this direction. Yet, we also highlight that from an epistemic perspective, the initial great promises that came with the introduction of omics research to soil microbial ecology were, after some time, increasingly doubted. Researchers then started to recognize various limitations of a solely omics-based approach to address the ecological questions of interest to them. These dynamics share parallels with developments in post-genomic research more broadly: The latter is sometimes seen as having overcome ‘gene-centrism’ and reductionism in the life sciences—whereas analyses now highlight that this change is not as complete as sometimes depicted and that ‘postgenomics continues to promise big, genes and sequences still play a central role in postgenomic thinking, and reductionism has not disappeared’ (Richardson & Stevens, 2015, p. 239). However, as we illustrate, the omics bandwagon in soil microbial ecology developed so much normative power that it has become difficult for researchers to re-orient their field away from it even though it is collectively desired to do so.

## Material and approach

This paper is based on an engagement with soil microbial ecology from three angles. First, we conducted an exploratory document analysis of publications from the field. The document analysis started from major journals in (soil) microbial ecology, focusing mainly on editorials and commentary pieces, and branched out from this using a snowball approach. The documents provided important background information for our analysis and informed subsequent empirical steps.

Second, we are involved in an ongoing collaboration with one soil microbial ecology research group in the project “Valuing-Being-Knowing” at the University of Vienna (01/2019-02/2024). We visited the group in the lab, had numerous informal conversations during observations of laboratory work and group meetings, and conducted fourteen interviews with researchers from the group. The qualitative, semi-structured interviews aimed at acquiring an in-depth understanding of the researchers’ epistemic agendas, of how their careers and epistemic foci have developed over time, and at mapping the various aspects that matter to them in making decisions in their work. Furthermore, researchers from the group were involved in two group discussions that centered around notions of innovativeness in the current research landscape, which equally provided relevant material for this paper.

Third, we conducted eighteen interviews, held online via video call, with researchers from the international soil microbial ecology community. The interviews focused on the development of the field, its central questions over time, and what factors had played a role in its development, with a particular focus on how researchers saw environmental questions, technological developments, and the conditions of the academic system as helpful to or hindering for their work. Six participants took part in a follow-up discussion that deepened some aspects explored in the individual interviews, and a couple of researchers engaged in further written conversations with us. Participants included current or former editors of major journals in the field, researchers who currently or previously had key roles in the International Society for Microbial Ecology [ISME], and individuals who wrote important publications in the field. Some participants were selected because they described their research agenda as particularly informed by concerns about social-environmental relevance. Besides identifying important figures ourselves, further relevant actors were indicated by our participants. Through our twofold selection strategy and the additional engagement with the local soil microbial ecology research group, our sample included both more junior and more senior, as well as more and less prominent researchers, which gave us some degree of diversity and breadth of perspectives on the field. In addition to the soil microbial ecologists, we interviewed another twenty-eight researchers working in soil-related research more broadly. While these interviews had a slightly different focus, they provided essential context for the dynamics described in this paper. Overall, participants came mainly from the Global North, and so our sample is mostly representative of Euro-American research cultures and academic systems.

Since we are interested in how researchers make sense of the developments in their field, the interviews and group discussions constitute the central material for our analysis. Our account of the subsequent re-orientations in soil microbial ecology is thus no neutral description of what is happening in the field. Rather, it is based on situated narrations that are influenced by researchers’ retrospective sense-making of the past (Bourdieu, 2017; Czarniawska, 2004) and by their current opinions and judgments about what is good research in soil microbial ecology. Researchers’ accounts of their current research practices and their future plans are situated in contemporary discourses about what it means to live and work in academia, such as for example on the importance of performing productivity and innovativeness in specific ways (Falkenberg, 2021). Our analysis is guided by a constructivist sensibility (Holstein & Gubrium, 1995) that is attentive to the ‘what’ (that is, how researchers choose their questions and approaches) as well as the ‘how’ of the terms in which they justify and make sense of these practices. Our long-term collaboration with one research group and repeated conversations with international researchers in different formats allowed us to analyze changes in research practices and how researchers discursively reflect on those changes in their field.

## Soil microbial ecology—central themes and epistemic challenges

(Soil) microbial ecology developed out of traditional microbiology in the second half of the twentieth century (Caumette et al., 2015; O’Malley, 2014). Particularly from the 1970s onwards, more and more ‘community-making devices’ (Molyneux-Hodgson & Meyer, 2009, p. 130) were established, such as textbooks in microbial ecology and journals like *Microbial Ecology* (1974), *FEMS Microbiology Ecology* (1985), or the *ISME Journal* (2007). Moreover, the International Commission for Microbial Ecology (now ISME) was founded in 1970 and has held regular symposia since the 1970s (Caumette et al., 2015).

Microbial ecology as such already combines skills and knowledge from various disciplines including microbiology, ecology, biochemistry, and bioinformatics. It is usually referred to as a discipline since it has established its own institutional structures such as journals and departments. Soil microbial ecology then may be considered a sub-discipline of microbial ecology, yet at the same time it also involves broader and partially very different skill-sets due to the specific complexities of soils and is institutionally less clearly circumscribed. We thus choose to refer to it as a field here.

Microbial ecology’s aim, in the words of practitioners, is to connect the ‘dynamics and composition of microbial communities with what they actually do in their natural environmental context’ (Hallin & Bodelier, 2020, p. 2; Kowalchuk et al., 2007; Veldkamp, 1985). As such, the discipline distinguishes itself from classical microbiological approaches. As one author puts it, microbial ecology is ‘not so much interested in the molecular biology or enzymology of microbial processes as […] in how these activities and the microbial populations that drive them act and interact in their environment’ (Kowalchuk et al., 2007, p. 2). At the same time, microbial ecology is also described as more mechanistically oriented than other kinds of ecology that are frequently portrayed as ‘just descriptive’ and more like natural history (Fierer, 2017; Hallin & Bodelier, 2020; Kowalchuk et al., 2007).

Initially, microbial ecology was rather marginalized within microbiology, which was dominated by a medical focus and by pure culture approaches, with comparably little interest in the environmental study of microbes (Caumette et al., 2015; O’Malley, 2014). This changed beginning in the 1980s with the influx of molecular technologies into microbial ecology. Today, technological innovation has ‘pushed the field to the forefront of microbiological research’ (O’Malley, 2014, p. 132). The discipline is thus often seen as technologically and scientifically ‘cutting-edge.’ However, as we will argue, researchers in soil microbial ecology now wish to orient their field away from a mode of research that is primarily technology- and data-driven.

Importantly, the focus on the study of *soils* seems to make dynamics in both epistemic re-orientations we describe somewhat more pronounced. Soils constitute extremely complex environments, characterized by great spatial heterogeneity and temporal variability (Fierer, 2017; Lehmann et al., 2020). On one hand, for a long time these complexities made it very difficult to study soil microbes at all, which facilitated the excitement about novel technological possibilities and the first re-orientation in the field. On the other hand, it is again the complexities of soils and a perceived need to also understand physico-chemical aspects studied by more traditional soil scientists that are now seen as necessitating broader interdisciplinary approaches to studying the functioning of soil microbial communities, thus fueling the demand for a second re-orientation.

Overall, the case of soil microbial ecology then provides an interesting opportunity: it allows to compare two rather different epistemic re-orientations, inquiring specifically into the role that innovation in research technology, packageability and signaling innovativeness, have been playing in constructing do-able problems and facilitating scientific change.

## First epistemic re-orientation: from cultivation to omics technologies

Studying the microbial ecology of soils is no easy endeavor, given the great temporal and structural heterogeneity of soils and the difficulties with making soil microorganisms ‘visible’. Until around the 1990s, soil microbial ecologists relied mostly on cultivation-based techniques. Yet, the cultivation of soil microbes was and is an extremely difficult, time-intensive, and risky endeavor—even today only a small fraction of soil microbes can be isolated and cultured. Hence, soil microbial ecologists could previously study only a few ‘laboratory rats’ (Prosser, 2019, p. 1), while the ‘uncultivated majority’ (Nesme et al., 2016, p. 2) was not accessible for investigation. As such, the field had long faced practical constraints on their ability to make do-able problems.

This changed considerably with the advent of molecular techniques for DNA analysis. Starting in the 1980s, successive advances in research technology enabled scientists to extract DNA from soils, analyze whole genomes, and eventually, metagenomics allowed them to sequence the genomes of microbial communities directly from environmental samples (Bertin et al., 2015; Caumette et al., 2015). In both the interviews we conducted and in publications, these developments are frequently portrayed as a ‘huge breakthrough’ (R44), as having led to ‘major advances’ (R53) or even a ‘revolution’ (R51) in soil microbial ecology (Caumette et al., 2015; Nesme et al., 2016). Early sequencing studies already indicated that soil microbial diversity was far larger than previously thought (Prosser, 2019) and particularly metagenomics and other subsequently developed omics approaches then allowed researchers to generate huge amounts of data about soil microbial communities which had been previously so difficult to study.

### Excitement, promising discoveries, and do-able problems

From both publications and our conversations, it seems that studies using molecular methods and omics technologies quickly came to dominate research in soil microbial ecology. While molecular techniques were developed in several steps that could be analyzed separately, our participants frequently grouped research using molecular omics techniques together when describing a particular ‘phase’ or mode of research in their field. They clearly distinguished this phase from previous research practices. Researchers recalled that this mode of research was seen as highly innovative, generating much excitement and promises for discoveries, and that it quickly became a relatively packageable approach. Both of these factors facilitated the construction of do-able problems.

Regarding the excitement it generated, it is important to note that the first re-orientation in soil microbial ecology did not take place in isolation. As one participant recalled, ‘DNA across the entire life science was … a game changer’ (R43), and the field was thus was riding on a broader wave of excitement, promise, and institutional support (see also Hilgartner, 2017). Yet, there seems to have been a specificity to these dynamics in the field of soil microbial ecology, which had previously been so limited in its epistemic possibilities due to problems posed by the great complexity of soils. As one participant put it:

We’re soil microbiologists who have known for many decades that plate counts only could pull up a few percent of the total number of cells in soil. We knew we were missing most of it—and now we can identify everyone? Wow! The field took to molecular tools like ducks to water. We could finally look at the things that defined our whole field! (R43)

One of their colleagues noted that ‘it was like the invention of glasses, now you can see everything… It was really a question of curiosity. Now we could discover all that stuff’ (R38). These quotations nicely illustrate the strong excitement going along with these novel technological possibilities and the discoveries they promised. Other participants also voiced the impression that because soil microbial ecology had been so constrained by the limited technological possibilities to investigate soil microbes, many soil microbial ecologists would, in turn, ‘go nuts’ for novel ‘tools for exploring microbes’ (R34) and generating large amounts of data about them—also because these data helped with ‘getting into the picture’ (R36), receiving attention, and making soil microbial ecology into an ‘acceptable field’ (R43).

Using molecular techniques also made it easier to get resources for studies in soil microbial ecology. It was easier to recruit new scientists, since ‘students are excited about new things… and new methods are often something they identify with. That was particularly true for the molecular methods’ (R42). It was also easier to acquire financial resources for molecular omics research. Especially as high-throughput sequencing and different omics techniques became more and more standardized—a transportable package that promised new discoveries—this package was ‘bought’ on different levels of research governance, from governments over research councils to peer reviewers. As one participant recalled, ‘there’s been pressure on research councils to increase omics research and that’s filtered down to the individual panels and so on’ (R49). Another researcher noted that ‘it seemed pretty quickly to become almost top-down driven that … major funding agencies began to, if not require, strongly encourage molecular analyses in the investigations’ (R52).

Molecular omics research not only allowed researchers to gain resources but also became a feasible pathway for ensuring productivity and building successful careers in soil microbial ecology. As participants described, many positions were created in this area and the prospects of a fruitful career following the molecular omics path even drew many people into the field who had been working in different areas beforehand. Furthermore, researchers described omics studies as a comparably safe path to productivity ‘because this whole genomics technology generates a lot of data with not so much effort. So, you go out spend some money, have a lot of data, and then a Ph.D. student is busy with that for the rest of the time, analyzing behind the computer’ (R33). While the interpretation of omics studies could still be challenging, researchers described the potential for data generation and the relatively straightforward problem-solution package (e.g., sequencing metagenomes from a particular environmental sample and describing the respective microbial communities) to be crucial to the traction this mode of research developed, especially after the field had been limited to risky and tedious cultivation work.

Overall, this mode of research was thus initially strongly appreciated in the research community and it relatively quickly became a fruitful way of creating do-able problems. This was facilitated through strong promises for discovery, as well as the packageable character of this mode of research—both going along with better possibilities for attaining resources and building successful careers.

### Bandwagon dynamics and their downsides

However, the re-orientation to molecular omics had some downsides for research in soil microbial ecology. The use of molecular techniques and particularly omics helped researchers create do-able problems (such as data-rich studies mapping the microbial community composition of a particular habitat) but over time became indispensable for doing so. Approaches such as cultivation or phospholipid fatty acid analysis—both established non-DNA-based approaches to analyzing microbial communities—were soon disregarded as old-fashioned. Similarly, measurements of processes and relevant environmental characteristics moved into the background. One participant recalled reading reviews on metagenomics, saying, ‘forget about everything anybody has ever known on soil microbial communities. You didn’t have metagenomics, so your data is worthless. You have to do metagenomics from now on’ (R49). Along these lines, researchers described that because omics were seen as promising and ‘sexy,’ and high-throughput data-rich approaches were valued so highly, they needed to use such approaches for getting funded and for publishing successfully. As one participant remembered:

During my Ph.D. and early Postdoc, I experienced one hype … when the sequencing technology suddenly enabled so much more analysis it started being applied to everything, and your grant or paper could be rejected just because you did not use the most up-to-date bioinformatic or sequencing methods. (R13)

These dynamics also played a central role in scientists’ future-oriented assessments of do-ability, as were especially apparent in narrations about junior researchers’ ability to make successful careers in the field. Participants recounted junior researchers’ worries about whether they could make a career in soil microbial ecology without being able to master molecular omics techniques, feeling a strong need to learn these.

These bandwagon dynamics impacted junior researchers more strongly than senior researchers in tenured positions, who sometimes noted that they had tried to circumvent the focus on molecular omics studies and stick to the broader ecological questions they considered relevant. Nevertheless, it seems that for a large part of the soil microbial ecology community, the bandwagon dynamics surrounding molecular omics studies developed an important formative effect on the choice of epistemic questions and approaches.

### Criticizing a focus on data-driven, descriptive science

Reflecting back on this history, many researchers also voiced doubts about the particular kinds of do-able problems addressed in omics studies. Participants pointed out that the rise of molecular methods and omics technologies led to a surge in primarily descriptive studies, cataloging microbes at the expense of hypothesis-driven research exploring microbial functions in ecosystems. As participants described, new techniques such as metagenomics were perceived as ‘a new frontier,’ meaning that ‘people are discovering things all the time.… There’s dozens and dozens of papers coming out.… But all the time people are describing, describing, describing’ (R35).

Researchers highlighted that the focal questions answered in molecular omics studies were mostly ‘who is there [and] what they have the capacity to do’ (R52)—i.e., ‘making lists’ (R52) of the microbes in a certain soil and inferring *hypotheses* about their *potential functions* but often not testing these hypotheses further. As participants pointed out, with DNA-based approaches alone, one could not go beyond this level and draw conclusions about the actual activity and ecosystem functions of soil microbes. Reflecting broader concerns in post-genomic research, which assert, as Russ Altman writes in the foreword to Richardson and Stevens (2015), that ‘determining the sequence of four DNA bases is easy … understanding its role in biological systems is [still] incredibly challenging’ (p. ix), many participants ultimately saw the potential of data-rich omics studies to be limited and the sole focus on such approaches to be problematic. As one researcher put it:

Even if we could have all the metagenomic data and all the databases with the gene knowledge, if we don’t have a good way to frame it, it’s not going to get us far… if it just stalls us out and we’re just distracted by data…. What we do with that will determine how much better we actually understand our systems (R34).

Overall, soil microbial ecologists were thus no longer content with the particular do-able problem-solution package created through molecular methods and omics approaches—they considered this mode of research as neither particularly relevant to social-environmental problems nor as helping to advance the science in their field.

## The wish for a second re-orientation: ‘Well-rounded’ studies

Against this background, nearly all of the researchers we talked to expressed enthusiasm for ‘a new generation’ (R43) or a ‘transition’ (R42) in soil microbial ecology, moving away from ‘the era of molecular methods’ (R42) and primarily descriptive studies towards a new mode of research that they considered as more scientifically and social-environmentally relevant, and better suited to deal with the complex interactions between microbial communities and their soil habitats. As one participant described:

We can now make lots of sequences… you have these huge surveys of microbial communities.… But now we’re moving to the next step, trying to… put the ecology part into the microbial ecology as opposed to now being really happy that we finally have tools to examine the diversity of these organisms…. Now we have these tools, what really makes the breakthrough is using them in a theoretical and experimental context. So now you don’t only use this huge omics toolbox to describe things in the environment. Now you actually do a dedicated experiment that looks at specific interactions (R44).

Along these lines, researchers noted that it was now important for soil microbial ecology to move beyond describing ‘who is there’, towards better understanding actual processes and ecological functions mediated by microbial communities such as ‘all the biogeochemical cycles … governed by microbes’ (R44) and to also investigate ‘when they do it, who is doing it, under which conditions’ (R12). When describing these encompassing studies they were wishing for, researchers often spoke of ‘well-rounded’ (R44) and ‘beautiful’ (R12) ‘back-to-back’ (R33) studies that investigate a certain ecological question from different angles to link microbial community composition to environmental functioning.

In terms of research practices, this re-orientation towards more ‘well-rounded’ studies entails two important aspects: first, studying not only genomes (and gene-products) but actual microbes, and second, investigating not only microbes but also their interaction with particular soil environments.

### Studying not only genomes but microbes too

Participants often noted that with the focus on data-rich omics studies, experimental studies looking at actual microbes and what they are doing had dropped out of the picture to some extent. Yet, researchers described a focus on actual organisms as indispensable to understanding how microbial communities participate in ecological processes such as carbon or nitrogen cycling. This sentiment parallels realizations in post-genomic research more broadly that ‘[t]he gene is not the organism’ (Fox Keller, 2015, p. 9). In other words, despite the successes of DNA (and RNA)-based approaches, understanding the role of an organism in ecological systems is still extremely challenging and necessitates more multifaceted research approaches (Richardson & Stevens, 2015). One participant described:

R36: That’s a general thing in science that a lot is going towards data, big data … but we should also … not lose the biology from it.… I don’t know why it sometimes gets so dis-balanced, but I guess it has to do with what’s cool, novel …

I: … and what does it mean to not lose the biology in this?

R36: Really the organisms and the way they behave and interact, because from soils we can collect DNA or even RNA and we can give names of what is in there … but really how they go about in the system, how they respond to each other, you don’t get that from a DNA or RNA.

Researchers thought that although omics and large-scale sequencing approaches were still seen as ‘cool,’ it was important for soil microbial ecology not to be limited to this way of creating do-able problems. When aiming to understand the actual activities and ecological functions of microbial communities—for example, their turnover of different substances such as nitrogen or carbon—it was indispensable to combine omics with a broader set of approaches. These could be other novel methods, such as modern mass spectrometry to analyze single cell functioning, isotope or imaging techniques, but also more traditional approaches, such as culturing microorganisms in the lab:

The funny thing is, microbiology comes from cultivation … and then there was this era of molecular biology and suddenly there were all these techniques, sequencing, PCR and so on…. with that, the attractiveness of cultivation has fallen and fallen and fallen … this went excessive, when we had these possibilities to sequence entire genomes simply from environmental samples. Also at conferences, for years … cultivation was not in the program anymore, it was really a science getting extinct. Then we realized that with sequencing we also run against a wall, this is all just potential … And suddenly cultivation is experiencing a revival (R12).

Many researchers asserted that to conduct the more ‘well-rounded’ studies they were aiming for, it was useful to go back to such ‘older’ approaches and combine them with other methods to integrate the different perspectives into a bigger picture about the ecological functioning of soil microbial communities.

### Understanding not only microbes but their interaction with particular soil contexts

Researchers further argued that ‘well-rounded’ studies should better understand the physical and chemical properties of the particular habitat in which microorganisms are situated. As noted before, soils are highly complex and characterized by great spatial and temporal heterogeneity. The characteristics of a particular habitat influence microbial activity and therefore the roles of soil microbes in ecological processes. For example, one researcher described that ‘the challenge with [the] carbon cycle is … often the physical and chemical environments regulate what the microbes can do’ (R43). Therefore, as another participant noted, ‘if you want to understand how soils work, you need to study soils—not just soil microbes, not just soil minerals but these are very complex and interwoven community processes where physical environment is as important as biological process’ (R34). While this need to study the abiotic aspects of soils together with the (micro)biological ones was now widely acknowledged, participants highlighted that soil microbial ecology had until now focused too little on how soil properties regulate and interact with microbial functioning. Sometimes, they explicitly related this neglect to the boom of the molecular methods:

With the growth of the molecular tools, there have been huge numbers of people working on soil organisms who… have never taken a course in soil formation, classification, soil chemistry. They don’t have that core background in how soil works. They’re great in extracting DNA and they can do anything with the DNA, but they miss what sometimes is really the controlling factor… Microbes may process organic molecules but what really is important in the long term is do microbes have access to those molecules. There are a lot of compounds in soil that are easily metabolized but might still have been there for a thousand years. It’s not that a microbe couldn’t eat it, it’s just a microbe never got it…If all you look at is the microbiology and the metabolic pathways, you’ll get cool papers, they might get published in Nature but they might be irrelevant actually explaining how something’s truly working in the field (R43).

Researchers who lacked this background now also recognized the importance of understanding at least some aspects of soil physics and chemistry. One participant noted that:

This is really about the interface … chemical, physical processes and what do my microorganisms do. I have to move more into that direction, either by doing literature research myself and trying to understand this, or by collaborating with other people. (R12)

The turn towards ‘well-rounded’ studies thus entails the integration of a broad range of technological and disciplinary approaches to study the ecological functioning of soil microbes in heterogeneous soil environments, rather than generating lots of data with one particular technique:

When we want to understand the functioning of these microbes in soil… you will never be able to understand and do everything on your own… Of course, you need to have microbiology but often you need to use stable isotopes. You need genomic techniques with all the bioinformatics associated to it. When you want to do it in a proper context, you need soil physicists…When it gets out of hand in terms of data, you need again bioinformatics staff. Basically, you will not be able to get to what you want to know without taking a lot of different disciplines on board. (R33)

This quotation nicely illustrates how extensive such ‘well-rounded’ studies can become and why these integrated studies are much less readily packageable than molecular omics studies centered around particular technologies.

### Well-rounded studies’ scientific and social-environmental relevance

Importantly, participants highlighted that such ‘well-rounded’ studies both were scientifically more interesting and made their field more relevant to current social-environmental crises. The previous omics studies were seen as relevant in so far as they helped soil microbial ecology bring their topics to political and societal attention and highlight the importance of protecting and better managing soil microbial communities (see also Granjou & Phillips, 2019). Yet, they were now considered as having, in themselves, limited value to implement these goals and further advance the field. Part of researchers’ motivation to move away from this bandwagon was that they saw the ‘well-rounded’ studies to be better suited for producing actionable knowledge for ecologically grounded applications such as climate change mitigation.

That this new mode of research is broadly appreciated in the soil microbial ecology community and that many researchers want to re-orient themselves away from the do-able problem-solution package of describing microbial communities using omics approaches indicates that researchers’ collective sense-making about more relevant modes of research can be an important impetus for change in research fields. Yet, while considerations about scientific and social-environmental relevance may play an important role, epistemic re-orientation also needs to be aligned with conditions for creating do-able problems. Indeed, despite the broad support for conducting more ‘well-rounded’ studies within the community, participants described various problems with making such studies do-able, which, until today, seems to hinder a full establishment of this new mode of research. We note that the problems with putting into practice the present re-orientation were not seen equally by all researchers. Some participants, mostly more senior ones with more flexible resources available, saw fewer problems with putting ‘well-rounded’ studies into practice. Yet overall, most researchers described tensions with re-orientating their field fully away from the bandwagon of data-rich omics research.

## Turning towards ‘well-rounded’ studies?

### Tinkering, riskiness, and uncertain time investments instead of ready-made packages

As described before, the ‘well-rounded’ studies researchers now desire entail the combination of various kinds of skills, technological approaches, and disciplinary expertise. They often require considerable time to set up collaborations, constant tinkering throughout the research process, and they are both risky and time-consuming. In contrast to the previous omics studies, they thus hardly come along in a readily packaged manner. This means that they cannot be easily assessed and supported by funders and that they do not necessarily ensure productivity and competitiveness. As such, they do not easily allow researchers to construct do-able problems in both the future- and the present-oriented dimension.

Participants frequently noted that third party funding was often indispensable for these studies, because even at comparably large and well-equipped institutions, ‘a lot of disciplines you don’t have on board so you need to find cooperating partners… you usually do that through project proposals’ (R33). Yet, these studies often didn’t fit well into competitive funding frameworks. Researchers reported that grants from national funders were relatively small in terms of grant size and duration, and so answering ecological questions about soil microbial functioning would usually require continued work over consecutive projects to establish an understanding of the community composition and experimentally test their functions and interactions within a certain habitat in different steps. Such continuous work on a certain topic, however, was difficult when researchers had to acquire new grants every three years. In the European context, large EU consortia could in principle fund these ‘well-rounded’ studies. Yet, researchers noted that ‘these big European calls’ (R38) were hardly an option for smaller institutions. Moreover, they saw these kinds of projects as focused on the ‘low-hanging fruits’ (R39) that were already very close to application, and not on projects combining application-oriented work with more fundamental questions. In specific national contexts, participants mentioned possibilities for establishing the collaborations necessary for conducting these studies through specific funding programmes which allow for coordinated interdisciplinary efforts to tackle specific research questions from different angles over a period of more than five years. Yet, within the current research funding schemes very few participants have the chance to be part of such longer-term initiatives.

In addition to the difficulties of attaining resources in the present, researchers also expressed concerns about remaining productive and building successful future careers when relying on this mode of research. Participants reported that ‘well-rounded’ studies that answer relevant ecological questions were appreciated by major journals for their scientific and social-environmental relevance, making these a seemingly good route for productivity. However, researchers highlighted that due to their less readily packageable character, ‘well-rounded’ studies would often require a longer time to be brought to a satisfactory end, which could be in tension with demands for publishing extensively and quickly. As one group leader elaborated:

I like to publish complete stories … coherent and from different perspectives, but then the doctoral student is there, who needs their three publications, and the [funding agency] wants to see in the final report that the whole thing has been productive. (R12)

This clash between the time required to bring ‘well-rounded’ studies to a satisfactory end and the need to show productivity within relatively short timeframes could become particularly problematic in the highly competitive field of microbial ecology.

Researchers thought that publishing ‘well-rounded’ studies would also be riskier. This was partially due to their complex interdisciplinary character but also because the different experimental approaches they entail can be more prone to failure, especially given the need to adapt these to heterogeneous soil environments. As one researcher put it, ‘a Ph.D. student here has four years. When you start cultivating an organism which you have no way of knowing beforehand how long it takes… it’s mainly too risky… it’s also difficult for short-term research to do it’ (R33).

Here, researchers saw a crucial difference between omics research and the desired ‘well-rounded’ studies. While the latter required considerable time, challenging interdisciplinary collaborations, and approaches such as cultivation work that are risky and likely to fail, researchers pointed out that the problem-solution package of generating omics data was a comparably safe approach. As one participant put it:

If all you’re saying is… in a year’s time I’ll have 10,000 sequences, five metagenomes and I’ll analyze them, in one way it’s safe. You’re going to get data. And as a Ph.D. supervisor, you relax when a student has some data. If at the end of the first year they’ve got nothing, you’re starting to worry for them and they’re starting to worry (R49).

### Difficulties in articulating novelty, innovativeness, and excitement

Next to these difficulties posed by the less packageable character of the ‘well-rounded’ studies, researchers also described that these often could not straightforwardly be turned into do-able problems because they were less easily recognized as ‘cool’ and innovative in the research system—thereby, again, making it more difficult to gain resources in the present or build successful careers with this mode of research. With regards to gaining resources for ‘well-rounded’ studies, participants described that:

One of the challenges of interdisciplinary work is if you’re trying to build a bridge, you don’t build it up on the very peak of the knowledge of each field.… You build that bridge a little bit down the shoulders where you know what you’re building on.… But that means that if either side reviews that proposal from its own perspective, it will never look cutting-edge because you’re not the most sophisticated within your own field. And that is one challenge … finding people who can actually acknowledge what you’re doing is truly new and thinking differently (R43).

In turn, researchers described that it could be easier to get funding for projects that are recognized as novel and ‘sexy’ across disciplines because ‘these kinds of things help [reviewers] understand more easily what you want to do’ (R33). This seems to have been a crucial advantage for the re-orientation towards molecular methods and omics technologies, as the latter were seen as promising tools across the life sciences and had a very ‘apparent potential for big new discoveries—we can first open a new black box. This is an easy sell even to non-experts’ (R43). Both of these characteristics seem to be rather lacking with regard to the ‘well-rounded’ studies.

Some participants saw it as easier to articulate the importance and exciting character of their work when employing high-throughput technology and focusing on generating a lot of data: ‘often it’s easier to get funding for those types of things, even though they’re not as smart science but they look really impressive’ (R44). Researchers suggested that because omics studies were aligned with a larger trend towards (big) data-intensive research, such studies would be favoured over ones that might, from their perspective, ask more thoughtful and potentially relevant ecological questions. Participants thus felt locked into the bandwagon they actually wanted to get away from.

Researchers also argued that it was easier to build successful careers with approaches that are well aligned with broader innovative trends (such as omics) instead of having a more interdisciplinary orientation. They thus saw the broad orientation of the ‘well-rounded’ studies as clashing with dynamics of the scientific system that encouraged ‘specialism’ and disciplinary work. As one participant put it, ‘there’s this tension if you’re building a new career, between how to do something cool and impressive that’s doable, which often requires carving things out very tightly and sharply vs. the more integrative [work required for well-rounded studies] and how do you juggle those different elements’ (R43). Some researchers perceived it as easier to make a career when fully committing to an approach seen as ‘cool’ more broadly, such as omics techniques, than with the multifaceted expertise required for ‘well-rounded’ studies.

## Conclusions

In this paper, we have explored how possibilities for creating do-able problems and researchers’ collective sense-making about desirable modes of research are intertwined in bringing about or impeding epistemic re-orientation in science. In order to do so, we have investigated two epistemic re-orientations in soil microbial ecology and discussed why researchers see the latter of these re-orientations as more difficult to bring about within the current academic system. We have shown that a first re-orientation towards molecular methods and omics techniques, although technologically challenging, took hold relatively smoothly because it soon allowed researchers to create do-able problems. The field is currently engaged in a second re-orientation towards encompassing ‘well-rounded’ studies that combine various disciplinary and technological approaches. Researchers see this mode of research as better suited to study the complex interactions of microbial communities with particular soil habitats and to increase the field’s capacity to address social-environmental problems. Yet, in contrast to omics studies, the desired ‘well-rounded’ studies are not easily turned into do-able problems as they are neither readily packageable nor as easily articulatable as innovative and exciting. Thus, although being collectively desired and considered more scientifically and social-environmentally relevant, epistemic re-orientation can be hindered by difficulties to turn a new mode of research into do-able problems—difficulties that may structurally favor particular kinds of re-orientation over others.

We see our paper as contributing to STS literature in different ways. While emphasizing the importance of possibilities for creating do-able problems in epistemic re-orientations, we have adopted a perspective that, in contrast to previous work (Fujimura, 1987, 1988), considers researchers not only as strategic actors who aim to create do-able problems but as also bringing other valuations to their work (see, e.g., Fochler et al., 2016; Sigl, 2019). This approach has helped us illustrate that it is not only do-ability that plays a role in re-orienting fields, but also collective sense-making about the desirability of new modes of research for reasons of scientific and social-environmental relevance. We thus underline the importance of taking into account various evaluative principles that researchers bring to their work when analyzing change in science in order to better understand how do-ability—as shaped by conditions of research governance—relates to aspects such as scientific and social-environmental relevance.

We have also suggested it is helpful to adapt and re-articulate the concept of ‘do-ability’ to better articulate how do-ability comes to matter in specific contexts of the research process to make the notion even more productive for analyzing change in the contemporary research system. We have therefore explicitly differentiated two temporal dimensions in researchers’ considerations about whether particular problems are do-able or not: first, present-oriented considerations about whether the material, human and temporal resources for conducting a particular research project are available or attainable and, second, future-oriented considerations about the potential of a research endeavor to support a successful scientific career through enabling productivity and competitiveness. We have also identified two cross-cutting aspects that make it easier to turn a new mode of research into do-able problems: the packageability (Fujimura, 1988; see also Kaltenbrunner, 2020) of a mode of research and its potential to signal innovativeness and novelty.

The degree to which a certain mode of research can be packaged plays an important role in facilitating or impeding epistemic re-orientation because it is crucial for attaining resources, being productive, and building successful careers—i.e., both the present and the future-oriented dimensions of do-ability. As we have illustrated, in the turn to molecular methods and omics research, a do-able problem-solution package based on a set of technologies was available and soon ‘bought’ by funding agencies riding the wider omics wave, allowing researchers to easily do this research in their funding environments. In contrast, the present ‘well-rounded’ studies are less readily packageable in any standardized manner. They lack a clear technological basis and often require considerable customization, tinkering, and time to establish large interdisciplinary collaborations, or to acquire interdisciplinary skills as an individual researcher. Such projects are more difficult to put into practice when dependent on short-term project grants. Moreover, in contrast to more readily packageable omics studies, the ‘well-rounded’ studies pose greater risk to publication-based productivity and are hence a less safe bet when it comes to building successful careers (a key concern for junior researchers in particular).

Next to packageability, we have focused on an aspect that has gained less attention in previous work on the role of do-ability in scientific re-orientation, namely on the degree of excitement that a certain new mode of research can create—its potential to function as an ‘emotional accelerant’ (Pickersgill, 2021, p. 600) and signal novelty and innovation across research fields (Falkenberg & Fochler, 2022). We have illustrated that novelty and innovativeness is crucial for enabling do-ability and hence for bringing about epistemic re-orientation. Our case suggests two factors contributing to whether new a new research mode might signal innovativeness. First, it seems to help if a mode of research can refer to a broader, already established bandwagon. Molecular methods and successive developments in omics technologies were part of a larger hype that went along with huge promises far beyond the field of soil microbial ecology (see, e.g., Richardson & Stevens, 2015), which was crucial in facilitating the first transition compared to the second. Although trends towards more systems-oriented approaches that align with researchers’ idea of ‘well-rounded’ studies have been described also in other fields, including systems biology, ecology, and soil science (Calvert & Fujimura, 2009), this mode of research does not have the same bandwagon character as molecular methods, omics, and big data. Second, new modes of research which construct a linear narrative relation between the novel approach and a frontier to be crossed are more readily seen as innovative, as was the case with the introduction of molecular methods in soil microbial ecology. In contrast, the sense of novelty going along with the ‘well-rounded’ studies is more complex. They aim to explore complex ecological interactions, and to build an understanding at the intersection of disciplines instead of pushing a clearly circumscribed frontier. Their novelty rather lies in producing new kinds of knowledge of higher social-environmental relevance and in developing more collaborative modes of creating knowledge. Because the ways in which frontiers are crossed in this modus are less tangible and the ways in which success is assessed are less standardized, ‘well-rounded’ studies seem to be much more difficult to establish as an exciting innovative program beyond the field of soil microbial ecology.

We believe that these insights into the roles of both packageability and innovativeness/excitement in epistemic re-orientation raise important implications for research governance. While the study of soils is specific insofar that soils are highly complex systems that pose great epistemic challenges to research, other research fields similarly aim to overcome genomic and post-genomic reductionisms (Richardson & Stevens, 2015) and to move toward more integrated and system-oriented modes of research (see, e.g., Calvert & Fujimura, 2009). Such transformations are often considered particularly important in light of current ‘wicked’ social-environmental crises (Brown et al., 2010; Kawa et al., 2021; Sigl et al., 2023). Yet, it is likely that other more integrated modes of research also lack the packageability and broadly recognized innovativeness that facilitates epistemic re-orientation. Therefore, if such re-orientations of research fields towards modes of research that allow for a better exploration of complex environmental relations are to be realized, it is important to attend to the affordances of the academic system. We acknowledge the increase in funding schemes and institutional arrangements that aim to foster more integrated modes of research, such as in recent EU research funding programs (European Commission, 2021). However, our analysis indicates that current funding conditions and dominant modes of research evaluation do not yet grant researchers enough flexibility and ‘protected spaces’ (Whitley et al., 2018) to seek out the collaborations necessary for more integrated research endeavors and to build such work over time. The research system still encourages approaches that allow for do-able problems to be constructed easily, which may produce an implicit bias towards ‘convenient’ data-driven (Krohs, 2012) and technology-focused modes of research.

Similarly, our analysis casts a somewhat critical light on discourses of radical innovation, ‘breakthrough,’ and ‘frontier’ science that are omnipresent in current research governance (Falkenberg et al., 2022; Flink & Kaldewey, 2018). Soil microbial ecology is presumably not the only field where a re-orientation is seen as crucial for fostering scientific and social-environmental relevance, but does not evidently promise ‘breakthrough’ discoveries or comes with an exciting technological bandwagon. As such, the rhetoric of cutting-edge science and fundamental ‘breakthroughs’ may delay scientific fields in maturing and in conducting research that can make relevant contributions to social-environmental problems. We thus see it as urgently necessary to more explicitly value and build capacities to recognize other forms of innovativeness in the research ecosystem.

Especially at times where science is increasingly called upon to produce knowledge relevant to pressing social-environmental problems, it seems rather worrisome if change in science remains strongly shaped by what has a good fit with current productivity- and competition-oriented conditions of research governance, or what falls in line with technological bandwagons and promises ‘breakthrough’ discoveries. Instead, we should work towards making room for a multitude of kinds of scientific creativity, innovativeness, and relevance. We believe that by delivering insights on the entanglements between epistemic developments and conditions of research governance, STS perspectives have an essential role to play in fostering such change.
